# Letter to the Editor Regarding: Evolving Business Models in Orthotics by Schneider, N.

**DOI:** 10.33137/cpoj.v5i2.38313

**Published:** 2022-12-31

**Authors:** C. Pardy, S. Scott, J. Barnert, C. Reimer

**Affiliations:** 1 Alberta Orthotic and Prosthetic Centre, Calgary, AB, Canada.; 2 Cascade Prosthetic Services, Calgary, AB, Canada.; 3 Colman Prosthetics and Orthotics, Calgary, AB, Canada.

**Keywords:** Orthosis, Prosthesis, Prosthetics, Orthotics, Funding, Economic, Health Care, AAOP, AOPA, AADL, Orthotist, Prosthetist, Rehabilitation, Mobility Impairment

## Abstract

The purpose of this letter is to continue the dialogue regarding the paper “Evolving business models in Orthotics” in the Canadian Prosthetics & Orthotics Journal Volume 4, Issue2, No.3, 2021. In it we present the perspective of the current Alberta Association of Orthotists and Prosthetists (AAOP) and provide additional context and information on historical events. Finally, we provide additional clarity on how costing is approached in the Province of Alberta (Canada) and the purported inequity in compensation between the two disciplines.

Dear Canadian Prosthetics & Orthotics Journal Editorial Board,

This response is on behalf of the Alberta Association of Orthotists and Prosthetists (AAOP). The AAOP is a voluntary non-profit professional organization that promotes communication and awareness in all matters pertaining to our profession. Within that framework, our role is to promote the professional services of our members within Alberta who provide orthotic and prosthetic treatments which enrich the lives of those we help. This role includes being a collective voice in discussion with various organizations with respect to matters of pricing and policy. We are not a governing body and do not set pricing on our own accord. We have a recommended pricing structure, though no individual clinic is required to adhere to it.

The purpose of this letter is to comment on events and actions attributed to our association contained within the recent CPOJ article “*Evolving business models in orthotics by Schneider, N*.”^1^ and provide accurate contextual information and insight into the pricing of orthotic and prosthetic treatment in Alberta in general, and how it is applied by Alberta Aids to Daily Living program specifically. We hope this will help to expand the discussions that are presently occurring across the country with respect to National and Provincial pricing guides.

To begin with, this article^1^ states that it provides a broad perspective of the orthotic business model and improvements that would have a positive impact on patient care and orthotic compensation. While that is a notable and welcome endeavour, in our opinion, we find the focus strays from that objective preferring to concentrate on grievances with the AAOP and a perceived discrepancy between prosthetic and orthotic compensation. It must be noted that this article^1^ focuses on pricing as it pertains to the Alberta Aids to Daily Living program (AADL) even though, they are one of many organizations that provide funding for orthotic and prosthetic care and treatment to Albertans with mobility impairment.

Orthotists and Prosthetists in Alberta are not licensed by the province and membership in the AAOP is voluntary and has no authority to dictate pricing or pricing models. Current AADL policy states that pricing is to be established through a “*mutually agreeable*” process, and in the past, AADL has worked with the AAOP to establish pricing. That is no longer the case as AADL sets its pricing with minimal consultation from the Vendor representatives. Each clinic has the freedom to establish its own pricing methodology for care and treatment of clientele who do not meet AADL eligibility criteria.

The overarching argument of this article appears to be that the Governance of AAOP has limited Orthotists and fostered a long-standing structural inequity between orthotic and prosthetic treatment. Further, that this perceived inequity is reflected in the AADL’s Approved Products Lists (APL’s) for orthotics and prosthetics,^2,3^ and favours prosthetics at the expense of orthotics. This document will address and provide documentation supporting the position we will put forth. The article^1^ lays out many points purporting to substantiate their argument, we would like to add additional information and provide a more accurate picture of orthotic and prosthetic pricing in Alberta in 2021. The underpinnings of the article’s inequity argument appear to stem from the following issues.

## Pricing of Orthotic Procedures in Alberta

The article^1^ stated that the current AADL’s Approved Product Lists are loosely based upon the American Orthotic Prosthetic Association’s (AOPA) cost accounting manual,^4^this is not entirely accurate. Prior to 2001 this was the case, however, since 2002 they have been based loosely on a formula developed by Dr. Phillip Jacobs in 2001, though there are some similar points in common with the AAOP cost accounting system.^5^

From 2002 through to 2017, AADL pricing has been based on the following formula, Price = direct materials (materials + 12% handling/loss/rework/warranty) + Shipping + (rate × time) this is substantially more closely aligned with the initial formula presented by Dr. Jacobs, price = direct materials + (rate × time) than it is with the AOPA cost accounting method.

This method is still in use for pricing most of the orthotic and prosthetic care however, AADL abandoned it for some of the procedures and new benefit codes introduced in 2017.

It is important to note that handling/loss/rework/ warranty “*markup*” has not increased since 2003 but, in fact, decreased from 16% to 12% with the implementation of the “*Merle Taylor*” report ^6^ of 2008. This markup does not vary from component to component as the paper^1^ suggests but is applied equally across components and disciplines.

## Times to Perform Orthotic Procedures

The perceived inequity if procedure times stems from an alleged 1991 AAOP decision to discount the times to perform orthotic procedures in the AOPA cost accounting system by 20%. No citation was provided for this claim other than a statement that this was reported by an AAOP member at an AAOP meeting on September 19, 2002, 11 years after that event. A search of the AAOP archives have revealed no discount occurred; personal communications with the individual specifically named, indicated that he has no recollection of making that statement, though he does recall the meeting.^7^

Further, discussions with Orthotist members who were practicing and active with the AAOP at the time have no recollection of this. However, they did indicate that the AADL, on their own initiative, did reduce the times of some knee bracing procedures and there is some debate as to whether that has been rectified with the current pricing methodology. There exists some evidence that this occurred but was limited in scope of application and appears to have been addressed in 2001 and 2002.^7^ Additionally, a scan of the times to perform orthotic procedures in Alberta and those of other jurisdiction that also used the AOPA cost accounting manual, as well as the Merle Taylor Report that reviewed selected times to perform procedures from Ontario and Manitoba show no evidence of any serious discrepancy in this area (Alberta Professions and Occupations Bureau Letter, Charlton D to Guest D, August 08, 1991; Ref: HDB.31/8974-1).

The standard mantra of documentation is that “*if it is not written it did not happen*”, in this case no written documentation is presented or can be found; therefore, one can only conclude that this did not happen.

## AAOP Introduced a Profit Margin on Material Costs

The article^1^ asserts that in 1991 the AAOP introduced a profit margin on material costs that varied from component to component, and that the original AOPA cost accounting system contained no such profit margin. A review of the AOPA cost accounting manual indicates that a “*loss and rework*” factor are added to the material and component costs prior to arriving at a “*total cost of direct materials*”. This varied from component to component based on risk of loss and formed the material cost entry on the calculations worksheet.^4^ It was this practice, consistent with the AOPA cost accounting system that was added into AAOP calculations and is not a margin of profit.

## Governance of AAOP

The article^1^ refers, though provides no citations, to three studies being instrumental in the development of pricing formulae: the study by Dr. Jacobs, the Frameworks Survey, and the Merle Taylor report. Both Dr. Jacobs study and the Merle Taylor report were funded by AADL, while the Frameworks study was funded by the AAOP. The Frameworks study did not validate Dr. Jacob’s work, as it dealt with times to perform procedures and not compensation or labour rates. After a thorough review the AAOP determined Frameworks contained some serious flaws and was never accepted by the AAOP and was not officially given to AADL and played no role in the pricing discussions. Ultimately, the AAOP committee decided that an enhanced version of the Dr. Jacobs formula would become the go forward pricing formula presented by the AAOP to the AADL in April 2003.^7^

There are no documents to support the argument that AADL has “*offered*” to implement separate applications of the AOPA cost accounting system. AADL consistently used the “*Merle Taylor*” formula from 2008 until 2017, when this was arbitrability changed by AADL without AAOP input. In 2007, a proposal was put forth to AADL to implement a pilot project creating a “*service*” model of pricing for Orthotics.^8^ This was never implemented. While the membership of the AAOP has fluctuated through the years the split between Orthotic and Prosthetic members has always been fairly even. At present AAOP has 35 members: 12-Prosthetists, 11-Orthotists, 6-Dually certified, 2-Associates (who are both from solely Orthotic facilities), 2-Registered Prosthetic Technicians, and 2-Registered Orthotic Technicians. It is not reasonable to assume that the Dual Certifees are partial to prosthetic issues over orthotic issues, nor is it reasonable to state that “*prosthetic issues tend to drive the agenda*”.

The article^1^ postulates that the governance practices of the AAOP limits orthotists. One example cited is inclusion in the Health Professions Act of 1996 and making the condition of licensure the minimum standard in Allied Health. Many factors work against licensure for this profession in Alberta and across the country and the AAOP has not been the only provincial association to attempt to achieve this goal. Issues that exist today were also present in 1996 and no provincial association has managed to attain licensure, however, in Alberta it has not been for lack of effort or acceptance of a government offer.

In 1991, the AAOP approached the Health Disciplines Board making a presentation on our profession. We were advised by the Registrar that the Act did not restrict the right to practice and only restricts the use of titles (Alberta Professions and Occupations Bureau Letter, Charlton D to Guest D, August 08, 1991. Ref: HDB.31/8974-1). At that time, subsequent the decision in the matter of Canadian Board for Certification of Prosthetists and Orthotists v. Canadian Pharmaceutical Association and Board for Orthotists Certification, titles were protected by trademark, and it appears the overriding opinion was this would have been costly and laborious for little to no gain. This matter was brought to the fore front by the AAOP, and the same individual who spearheaded the initiative in 1991, sought a legal opinion on this again in 2001. It was the opinion of the association’s lawyer (according to a letter by Renouf S to Guest D in September 27, 2001) at the time that success would have been unlikely. This was not simply a matter of the AAOP “*declining*” an offer.

## The Merle Taylor Formula

In response to an impasse in discussions, and a rejection of the pilot project for two different pricing methods for orthotics and prosthetics, the AAOP and AADL mutually agreed to a 3rd party review of orthotic and prosthetic business arrangements in Alberta. AADL contracted, and paid, Merle Taylor consulting to carry out this review. The objectives being to determine:

- Whether AADL’s current payment arrangements and fee schedule for orthotic and prosthetic services are adequate compared to similar services and
- Whether there are different payment approaches that would improve the efficiency and fairness of the payment process.

The result was a process that removed the aspect of profit from materials and componentry and created a formula for developing a charge-out rate (should never have been called a labour rate) that was equitable, though not equal, between orthotics and prosthetics. This formula takes into account public sector salaries for both clinicians and technicians, administrative time, inflation, billable/non-billable hours, benefits, weighted labour times (prosthetic more clinician time vs orthotics more technician time), overhead, and a profit objective. In short, all the costs of operating a prosthetic and orthotic facility are captured in this rate, as a result, the rates differ between orthotics and prosthetics but are equitable. The labour component of this formula was based on the “*top-of-range*” from the public facilities for both clinicians and technicians as derived from the Health Sciences Association of Alberta collective agreements and applied equally to both the orthotic and prosthetic calculations. They were not, however, based on “total compensation” as asserted in the paper.^1^

In 2008, the Merle Taylor Report determined that for facilities to realize a potential profit of 10% charge-out rates of $162.00/hr for prosthetics and $130.00/hr for orthotics was required. While the final numbers were different, these results were similar to internal reviews taken in 2002 that identified differing rates for orthotics and prosthetics. While this was equitable, it was not equal. It was put forward that given orthotists and prosthetists have the same qualifications and must meet the same national standards, the practice of blending the rates to create a single charge-out rate would continue. This reduced the prosthetic rate and profit potential and increased the orthotic rate and profit potential.

In 2010, AADL offered to discuss the separation of rates, while two attendees at the table agreed, the AAOP was unanimous keeping the same single charge out rate. The blending of the rates continues today, the current AADL charge-out rate that was established in 2019 of $185.77/hr^9^ which is a blend of the calculated prosthetic formula rate of $205.94 and the orthotic formula rate of $165.59, this is a direct subsidy of $20.18 favouring orthotics and changes the potential for profit to 1% for prosthetics and 25% for orthotics.

The article^1^ comments on subjects that are out of the scope of the AAOP, and which have been eloquently addressed in a letter to the editor by Orthotics Prosthetics Canada (OPC).^10^

In summary, the article^1^ in question did not provide a complete historical account of the development of pricing models for orthotic and prosthetic services used today with the AADL in Alberta. This letter aims to provide additional information and clarification on the opinion that prosthetics unfairly benefits at the expense of orthotics. It also provides the background of the development of our current pricing methodology and provides facts surrounding licensure.

There is a threat that exists for Albertans with mobility impairment, coming from the blended rate and removing profit potential from prosthetics. As AADL APL’s fail to keep pace with material inflation, more of the costs will fall on the clients who can least afford it. The ability to respond to articles such as this one^1^, providing additional perspective, is important so that individuals in positions of authority in matters such as this can base their decisions on a complete spectrum of available information in order to prevent further erosion patients’ accessibility to life enhancing care and treatment.

## CALL TO ACTION

While the article^1^ in question was, in our opinion not fully comprehensive it did broach a topic that merits more consideration. As Orthotists and Prosthetists, we need to continue to move away from being perceived to be providers of things, to the reality that we provide professional services to preserve our unique niche within the healthcare continuum. This has long been a goal of our national body, Orthotics Prosthetics Canada, who have been working hard on this transition with the ongoing changes to exams and the national body itself with great success. A national coding guide that furthers this transition is the next logical step and merits discussion and action.

Pricing for orthotic & prosthetic care and treatment is a complicated matter as we function in an area of Provincial Jurisdiction, also interacting with Federal Programs and nationwide insurance providers. Some provinces have established independent living programs that dictate pricing and service, but this is not universal in pricing formulae or benefits provided. Further, we provide professional services to support individuals with mobility or physical impairment, but at the end of the day it is a device that enables these individuals to attain a level of restored function.

The AAOP pricing manner discussed in the article^1^ and this letter may not be perfect, but the structure does represent a substantial move away from device-based model to a professional services model as it is clear and transparent with all the pricing inputs. Our understanding is that OPC has initiated the process of developing a coding document that can be a reference for the provincial associations and to that end, AAOP is putting our full support behind it.

## DECLARATION OF CONFLICTING INTERESTS

Connor Pardy is currently Past-President of the Alberta Association of Orthotists and Prosthetists (AAOP), and was the President at the time of writing. Connor Pardy is currently on the Board of Directors of Orthotics Prosthetics Canada (OPC), and is a practicing Certified Prosthetist and Orthotist at Alberta Orthotic and Prosthetic Centre.
